# Physical rehabilitation improves muscle function following volumetric muscle loss injury

**DOI:** 10.1186/2052-1847-6-41

**Published:** 2014-12-19

**Authors:** Amit Aurora, Koyal Garg, Benjamin T Corona, Thomas J Walters

**Affiliations:** Department of the Army, Extremity Trauma and Regenerative Medicine, Institute of Surgical Research, 3650 Chambers Pass, JBSA Ft Sam, Houston, TX 78234-7767 USA

**Keywords:** Muscle, Trauma, Rehabilitation, Running, Function

## Abstract

**Background:**

Given the clinical practice of prescribing physical rehabilitation for the treatment of VML injuries, the present study examined the functional and histomorphological adaptations in the volumetric muscle loss (VML) injured muscle to physical rehabilitation.

**Methods:**

Tibialis anterior muscle VML injury was created in Lewis rats (n = 32), and were randomly assigned to either sedentary (SED) or physical rehabilitation (RUN) group. After 1 week, RUN rats were given unlimited access to voluntary running wheels either 1 or 7 weeks (2 or 8 weeks post-injury). At 2 weeks post-injury, TA muscles were harvested for molecular analyses. At 8 weeks post-injury, the rats underwent *in vivo* function testing. The explanted tissue was analyzed using histological and immunofluorescence procedures.

**Results:**

The primary findings of the study are that physical rehabilitation in the form of voluntary wheel running promotes ~ 17% improvement in maximal isometric torque, and a ~ 13% increase in weight of the injured muscle, but it did so without significant morphological adaptations (e.g., no hypertrophy and hyperplasia). Wheel running up-regulated metabolic genes (SIRT-1, PGC-1α) only in the uninjured muscles, and a greater deposition of fibrous tissue in the defect area of the injured muscle preceded by an up-regulation of pro-fibrotic genes (Collagen I, TGF-β1). Therefore, it is plausible that the wheel running related functional improvements were due to improved force transmission and not muscle regeneration.

**Conclusions:**

This is the first study to demonstrate improvement in functional performance of non-repaired VML injured muscle with physical rehabilitation in the form of voluntary wheel running. This study provides information for the first time on the basic changes in the VML injured muscle with physical rehabilitation, which may aid in the development of appropriate physical rehabilitation regimen(s).

## Background

Volumetric muscle loss (VML) is the traumatic or surgical loss of skeletal muscle due to explosive munitions, bullet wounds, or surgical excision of a sarcoma with resultant functional impairment [1]. The indiscriminate nature of these insults results in the loss of myofibers, their associated satellite cells, other resident cells, basal lamina as well as intramuscular neural and vascular structures [2–7]. Following injury, the remaining muscle undergoes continued damage, develops fibrosis, and likely has gross architectural alterations. These changes are presumed to be the result of the initial injury and subsequent chronic overload on the remaining muscle as it attempts to compensate for the loss of a portion of the muscle.

Currently, there is no defined surgical standard of care for VML injuries. Clinically, these wounds are often surgically repaired with a fascio-cutaneous and/or muscle flaps. Importantly, these procedures are not intended to restore muscle function. The last decade has seen significant advances in the development of tissue engineering strategies for VML repair; although the clinical utility of these therapies is not yet realized [3–6, 8–11]. Hence, physical rehabilitation is the only therapeutic strategy for VML injuries, at least in the military medical system [2, 12]. However, physical rehabilitation is aimed at strengthening the remaining injured muscle, but not at promoting muscle regeneration.

Physical rehabilitation has been investigated as a strategy to treat acute muscle injuries (e.g., contusion) [13], for the recovery of skeletal muscle damaged due to age [14–16], pathological (e.g., muscular dystrophy), and metabolic (e.g., diabetes) conditions [17, 18]. For acute muscle injuries, it has been shown to accelerate muscle healing/ regeneration by modulating the immune response, facilitating vascularization and the release of pro-myogenic growth factors, and reducing fibrosis [19–23]. In contrast, the results of pre-clinical and clinical studies using physical rehabilitation to treat skeletal damage due to pathological conditions have been mixed. A few have reported on its benefit to maintain muscle strength [24] and reduce susceptibility to contraction-induced injury [25]. While others have reported it to cause strain injuries [26, 27], to be detrimental to muscle function [28], and/or to have no effect [29].

Unlike these muscle injuries and pathological conditions, VML injuries involve the frank loss of muscle tissue with concomitant damage to intramuscular neural and vascular structures. Hence, there is a need to understand the response of VML injured muscle to physical rehabilitation. Given the clinical practice of prescribing physical rehabilitation for the treatment of VML injuries, understanding the basic responses of the injured muscle to increased activity may aid in the development of appropriate rehabilitation regimen(s). The specific objectives of this study were to examine the functional and histomorphological adaptations in the VML injured muscle to physical rehabilitation. This was performed using an established rodent tibialis anterior muscle VML injury model [5, 7] and voluntary wheel running as model for physical rehabilitation.

## Methods

### Experimental design

A VML injury was created in the tibialis anterior (TA) muscle of thirty two adult male Lewis rats (3-4 months old; 325-350 grams; Harlan Laboratories, IN, USA) as previously detailed [5–7]. The rats were then assigned to either sedentary (SED) or physical rehabilitation (RUN) group and returned to individual cages (n = 8/group). After 1 week, RUN rats were transferred to individual chambers equipped with voluntary running wheels (Lafayette Instrument Company, Lafayette, IN, USA) and allowed unlimited access to the wheel for the either 1 or 7 weeks (2 or 8 weeks post-injury). At 2 weeks post-injury, TA muscles were harvested for molecular analyses. At 8 weeks post-injury, the rats underwent *in vivo* function testing as previously described followed by tissue harvest [5].

### Animals

This study was conducted in compliance with the Animal Welfare Act, the Implementing Animal Welfare Regulations, and in accordance with the principles of the Guide for the Care and Use of Laboratory Animals. All procedures were approved by the IACUC at the U.S. Army Institute of Surgical Research. Rats were housed in a vivarium accredited by the Association for Assessment and Accreditation of Laboratory Animal Care International.

### VML injury model

The surgical procedure for creating VML in the rat TA muscle was performed as described previously [5–7]. Briefly, using aseptic technique, a surgical defect was created in the middle third of the TA muscle using a scalpel. The excised defect weight approximated ~ 20% of the estimated TA muscle weight.

### *In vivo*functional analysis

The isometric contractile properties were determined *in vivo* on anesthetized animals as previously described [5]. The foot of the animal was strapped to a footplate attached to a dual-mode muscle lever system (Aurora Scientific Inc., ON, Canada), and the knee and ankle positioned at right angles. Body temperature was maintained at 36 - 37°C. Functional properties were first determined on the intact anterior crural muscles, followed by on the isolated TA. Isolation of the TA was accomplished by tenotomizing the extensor digitorum longus (EDL) and extensor hallucis longus muscles above the retinaculum, while keeping the tendon associated with the TA muscle including the retinaculum undisturbed. Maximal isometric torque (T_max_) was determined by stimulating the peroneal nerve using a Grass stimulator (S88) at 150 Hz with a pulse-width of 0.1 ms across a range of voltages (2-8 V).

### qRT-PCR

RNA was isolated from snap frozen cross sections of TA muscle that included the defect area and the remaining muscle (50-100 mg) and reverse transcribed to make cDNA. Aliquots (2 μL) of cDNA were amplified with 200 nM forward/reverse primers, SYBR GreenER (Life Technologies, NY, USA) in triplicate using a Bio-Rad CFX96 thermal cycler system. Non-template control and no reverse transcriptase controls were run for each reaction. Gene expression was normalized to 18S (housekeeping gene) to determine the ΔCT value. Expression levels for mRNA transcript were determined by the 2^-ΔΔCT^ method by normalizing each group to the uninjured muscle of the SED group [5]. Primer sets were synthesized by Sigma-Aldrich DNA oligos design tool (Table 1).

**Table 1:** **Nucleotide sequences of primers used for qRT-PCR**

Gene	Forward sequence	Reverse sequence	Amplicon length (BP)
**eMHC**	5′- TGGAGGACCAAATATGAGACG-3′	5′-CACCATCAAGTCCTCCACCT-3′	180
**Collagen-1**	5′-GACCAATGGGACCAGTCAGA-3′	5′-CTGGTGAACGTGGTGCAG-3′	123
**TGF-β1**	5′-GTCAGACATTCGGGAAGCA-3′	5′-CCAAGGTAACGCCAGGAAT-3′	138
**SIRT-1**	5′-GTTGACCTCCTCATTGTTATTGG-3′	5′-CGCAGTCTCCAAGAAGCTCT-3′	151
**PGC-1α**	5′-CGTGTTCCCGATCACCATA-3′	5′-GTGTGCGGTGTCTGTAGTG-3′	108
**18S**	5′-GGCCCGAAGCGTTTACTT-3′	5′-ACCTCTAGCGGCGCAATAC-3′	173

### Histological and immunofluorescence procedures

TA muscles were embedded in a talcum-based gel and snap frozen. Sections (~8 μm thick) were stained with hematoxylin and eosin H&E) [6]. Immunofluorescence stained tissue sections (~8 μm thick) were probed for collagen I (1:500; EMD Millipore Corporation, MD, USA), sarcomeric myosin (MF20; 1:10; Development Studies Hybridoma Bank, IA, USA), and nuclei (DAPI; 1:100; Life Technologies, NY, USA) [6]. Sections were blocked in 5% goat serum for 1 hour at room temperature and then incubated with primary antibody overnight at 4°C. Sections were then incubated in corresponding AlexaFluor 488/596 labeled secondary antibodies (1:200-1:500) for 1 hour, stained with DAPI and mounted. Qualitative assessments were made by observing three sections from 3 - 5 muscles per group.

### Quantification of centrally located nuclei

The total number of centrally located nuclei (CLN) were determined from H & E stained sections of uninjured and injured muscles (n = 6/group). Fifteen non-overlapping 100× images were taken from the superficial, middle, and deep regions of the muscles. The percent of the total number of CLN was obtained by normalizing number of CLN counted to the total number of fibers per image.

### Quantification of intramuscular collagen

The area fraction of collagenous tissue exclusively within the remaining muscle (not in the defect area) was determined from collagen I stained sections of uninjured (n = 3/group) and injured muscles (n = 6/group). Fifteen non-overlapping 100× images were taken from the superficial, middle, and deep regions of the muscles. The images were converted to 8-bit, background subtracted and rescaled if necessary from 0 (pixel with value of 0 is white) to 255 (pixel with value of 255 is black) before a threshold was applied to each image in Image J.

### Morphological analysis

Individual fiber cross sectional area (CSA) were determined from collagen I stained sections of uninjured and injured muscles (n = 6/group). Fifteen non-overlapping 100× images were captured from each muscle, and measurements were manually obtained using Image J. Only fibers between 50 and 8000 μm^2^ were included in the analysis [30]. The frequency distribution of fiber CSA was computed from individual fiber CSA measurements. Fiber counts were obtained by manually counting the number of muscle fibers using Image J from scanned H & E sections of the entire muscle (n = 5-6/group).

### Statistical analysis

Dependent variables were analyzed using a one-way ANOVA or independent samples *t*-test. Statistical significance was achieved at an alpha of 0.05 set a priori. Values are means ± SEM. Statistical testing was done with Prism 5 (GraphPad, La Jolla, CA).

## Results

### Wheel running

**Figure 1:**
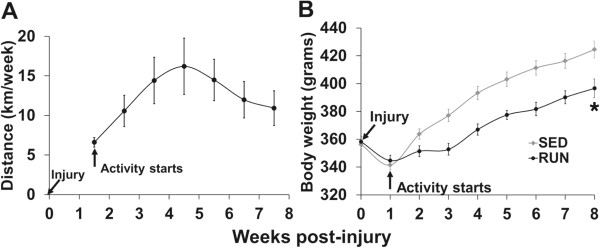
**Wheel running animals gained less weight throughout the study.** A subset of the animals was given access to voluntary running wheels one week post-injury and was allowed to run for 7 weeks **(A)**. At the end of 7 weeks, the animals in the RUN group were significantly (~10%) lighter than animals from the SED group **(B)**. * ≠ SED; p < 0.05.

All animals ran an average of 12 ± 1 km/week for 7 weeks. Running increased during the first four weeks, and then tended to decrease thereafter. The distance was significantly higher at all-time points compared to the first week (Figure 1A) (p ≤ 0.01). The maximum distance (16 ± 4 km) was comparable to that reported by Rodnick et al for rats in the low-activity group (14 - 35 km/week) [31].

### Body weight

Despite similar mean body weights (BW) prior to injury, RUN animals gained significantly less weight throughout the study (Figure 1B). At the end of the study, RUN animals were ~ 10% lighter than the SED animals (Table 2) (p = 0.02). Due to differences in BW, muscle weight and T_max_ were normalized to BW for statistical comparisons.

**Table 2:** **Body and muscle weight measurements**

	SED		RUN	
Parameters	Uninjured	Injured	Uninjured	Injured
**Sample size**	7		7	
**Body Weight at sacrifice (g)**	424 ± 7		397 ± 7^£^	
**TA Muscle weight (mg/g)**	1.68 ± 0.03	1.35 ± 0.03^*^	1.70 ± 0.03	1.52 ± 0.04 ^§*^
**EDL Muscle weight (mg/g)**	0.41 ± 0.001	0.50 ± 0.001^*^	0.43 ± 0.001	0.47 ± 0.001^*^

* ≠ uninjured (contralateral); § ≠ sedentary injured; £ ≠ sedentary. Values are mean ± SEM; p < 0.05.

### Muscle weight

The TA weight of the injured limb in either group was significantly less than the respective uninjured (contralateral) muscles (p ≤ 0.001) (Table 2). The TA weight of the injured limb from the RUN group was ~13% heavier than that of the SED group (p ≤ 0.01). The EDL weight of the injured limb in the RUN group was 9% higher than that of the uninjured limb (p ≤ 0.01). In contrast, the EDL weight of the injured limb from the SED group was ~ 22% higher than the uninjured limb (Table 2) (p ≤ 0.001).

### *In vivo*isometric strength

**Figure 2:**
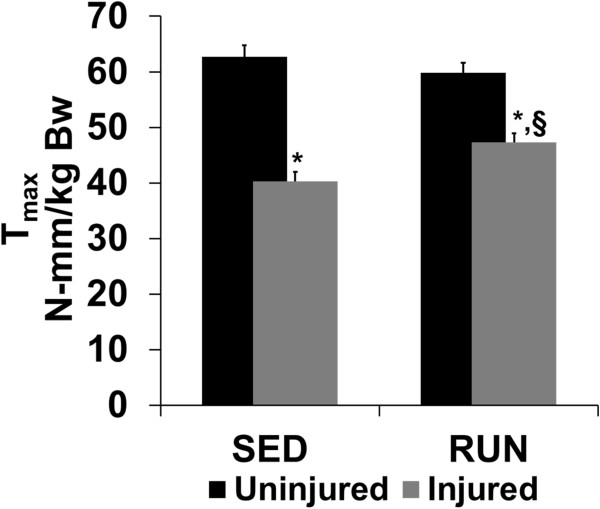
**Physical rehabilitation in the form of voluntary wheel running improves in vivo tibialis anterior muscle torque.** Maximal isometric torque (@ 150 Hz) of the tibialis anterior muscle was assessed *in vivo* following distal extensor digitorum longus muscle (EDL) tenotomy (see Methods). Average maximal isometric torque normalized to body weight is shown for the uninjured and injured muscle for the SED and RUN groups. Values are mean ± SEM. Sample size is listed in Table 3. * ≠ uninjured (contralateral); § ≠ sedentary injured; p < 0.05. All VML responses, regardless of group, were lesser than uninjured contralateral values.

**Figure 3:**
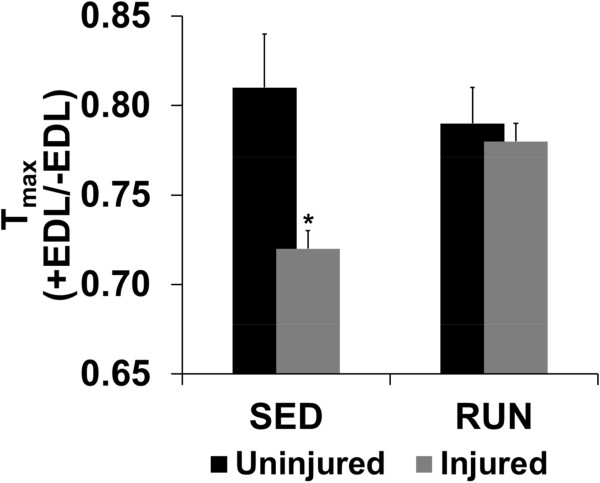
**Physical rehabilitation in the form of voluntary wheel running mitigates force imbalance developed as a result of VML injury.** Maximal isometric torque prior to tenotomy of the EDL was normalized to the maximal isometric torque after tenotomy of the EDL. Values are mean ± SEM. Sample size is listed in Table 3. * ≠ uninjured (contralateral); § ≠ sedentary injured; p < 0.05.

Prior to EDL tenotomy, T_max_ of the uninjured and injured anterior crural muscle was similar between groups, respectively (Table 3). VML injury produced a significant deficit of 25% and 20% in the SED and RUN group, respectively (Table 3, p ≤ 0.001). After tenotomy, the T_max_ of the isolated TA of the injured muscle in the SED and RUN group was 35% and 20% lower than the uninjured muscle, respectively (p ≤ 0.001, Figure 2; Table 3). The injured muscle in the RUN group generated 17% greater T_max_ than the SED group (p ≤ 0.01). In order to determine the imbalance in force created due VML injury T_max_ prior to EDL tenotomy was normalized to T_max_ after tenotomy. VML injury created a 12% imbalance in force, which was mitigated with wheel running (p ≤ 0.001) (Figure 3, Table 3).

**Table 3:** ***In vivo*** **contractile properties**

	SED		RUN	
T _max_	Uninjured	Injured	Uninjured	Injured
**Anterior Crural Muscles (+EDL)**				
***Nmm/kg body weight***	76.5 ± 2.1	55.8 ± 1.8^*^	76.1 ± 1.9	61.0 ± 2.4^*^
**TA Muscle (-EDL)**				
***Nmm/kg body weight***	62.7 ± 2.0	40.3 ± 1.7^*^	59.8 ± 2.0	47.3 ± 1.6^*,§^
**EDL Muscle**				
**T**_**max**_ **(+EDL/-EDL)**	0.81 ± 0.02	0.72 ± 0.01^*^	0.79 ± 0.01	0.78 ± 0.01

* ≠ uninjured (contralateral); § ≠ sedentary injured. Values are mean ± SEM; p < 0.05.

### Morphological analysis

**Figure 4:**
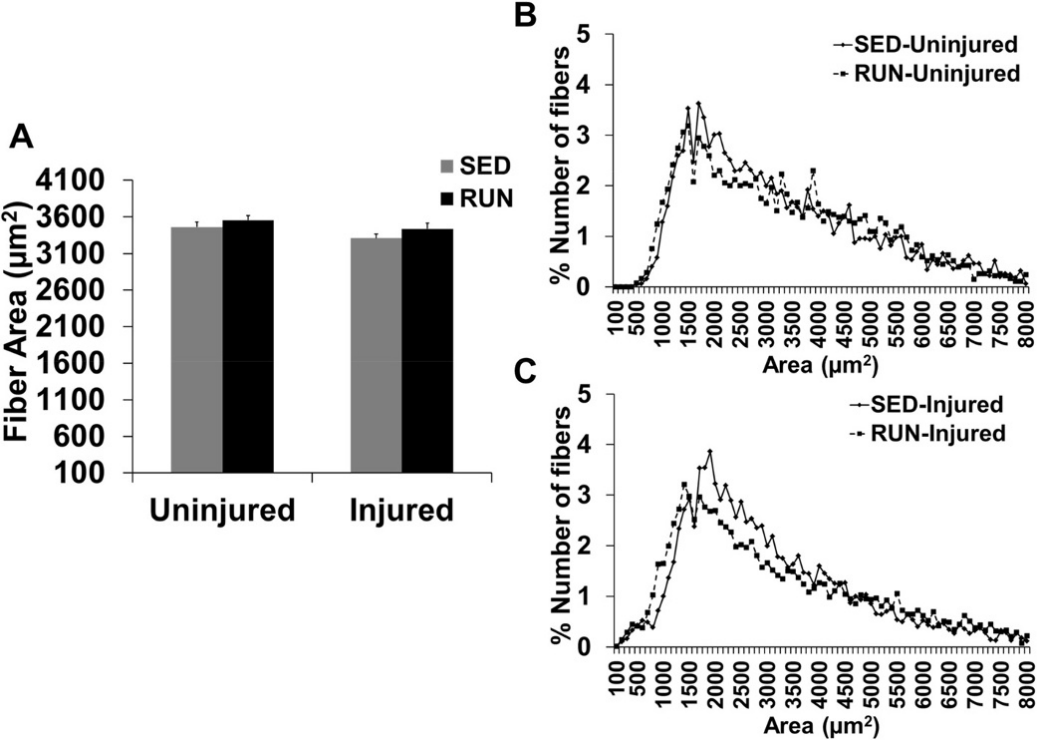
**Physical rehabilitation in the form of voluntary wheel running does not result in morphological adaptations (fiber cross-sectional area).**100× non-overlapping images from the injured muscle were analyzed for fiber cross-sectional area (CSA) measurements **(A)**. From these measurements, the fiber cross-sectional area CSA frequency distribution was obtained for the uninjured **(B)** and injured muscle **(C)** Values are mean ± SEM. n = 6 muscles/group; p < 0.05.

The muscle fiber cross-sectional area (CSA) including the frequency distribution profiles of the uninjured and injured muscle was similar between groups (Figure 4A-C). The total number of fibers in the injured muscle was ~35% lower than uninjured muscle, but there were no differences between groups (Table 4).

**Table 4:** **Morphological adaptations**

	SED		RUN	
Parameter	Uninjured	Injured	Uninjured	Injured
**Fiber CSA (μm** ^**2**^ **)**	3271 ± 49	3093 ± 47	3324 ± 52	3215 ± 48
**Total fiber number**	8458 ± 400	5772 ± 446	9665 ± 767	5970 ± 671

Values are mean ± SEM.

### Qualitative histological assessment

**Figure 5:**
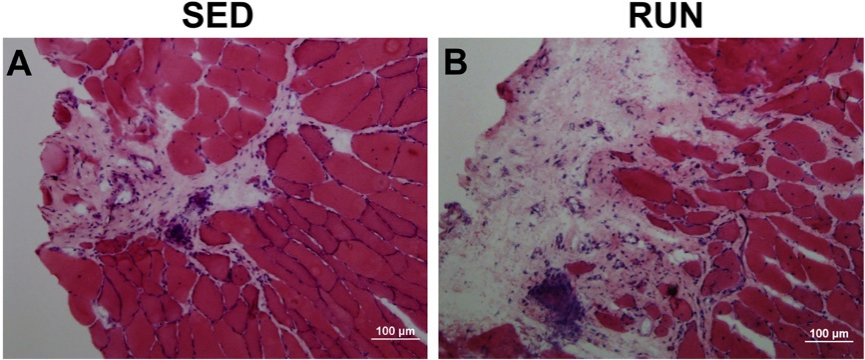
**Physical rehabilitation in the form of voluntary wheel running prevents collapsing of muscle fibers.** The muscle fibers collapse around the injury site in the SED group **(A)**, while they enclose the fibrotic scar in the RUN group **(B)**. In either group, the area immediately adjacent to defect has disorganized muscle fibers. Scale bar = 100 μm.

**Figure 6:**
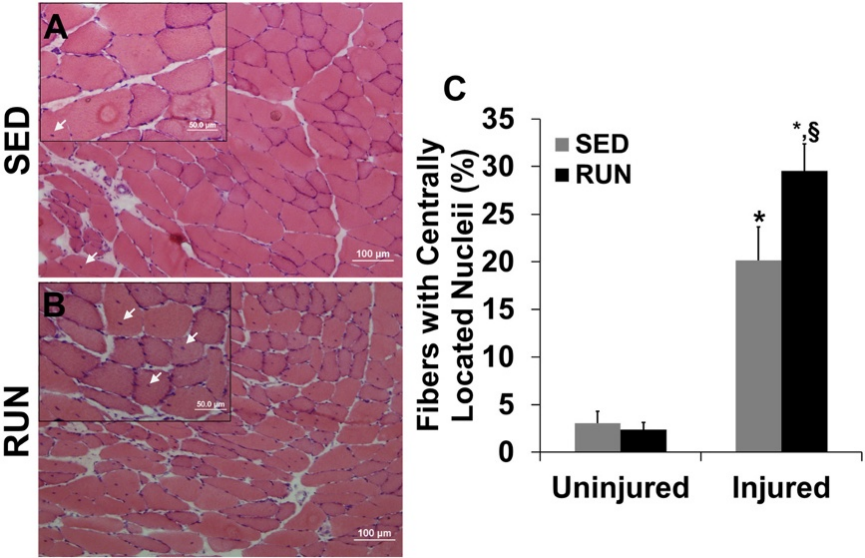
**Physical rehabilitation in the form of voluntary wheel running exacerbates chronic injury in the injured muscle.** Uninjured contralateral (not shown) and injured muscle of the SED **(A)** and RUN **(B)** groups were analyzed for the presence of centrally located nuclei (white arrows) (Scale bar = 100 μm). Inset images are high magnification (200×) images in the injured muscle (Scale bar = 50 μm). Physical rehabilitation significantly increased the presence of CLN in the injured muscle **(C)**. Values are mean ± SEM. n = 6 /group; * denotes ≠ uninjured (contralateral); § denotes ≠ sedentary injured; p < 0.05.

A fibrotic scar was formed in the defect area in either group, which was more pronounced in the RUN group (Figure 5A-B). The muscle fibers appeared to collapse around the injury site in the SED group (Figure 5A), while they enclosed the scar in the RUN group (Figure 5B). In either group, the area immediately adjacent to the defect contained disorganized muscle fibers radiating inward from the injury site with evidence of fiber damage noted by the presence of CLN (Figure 6A-B). The injured muscle in either group had significantly more fibers containing CLN than the uninjured muscle. The injured muscle in the RUN group has ~50% more fibers with CLN than the SED group (Figure 6C) (p ≤ 0.04).

### Intramuscular collagen

**Figure 7:**
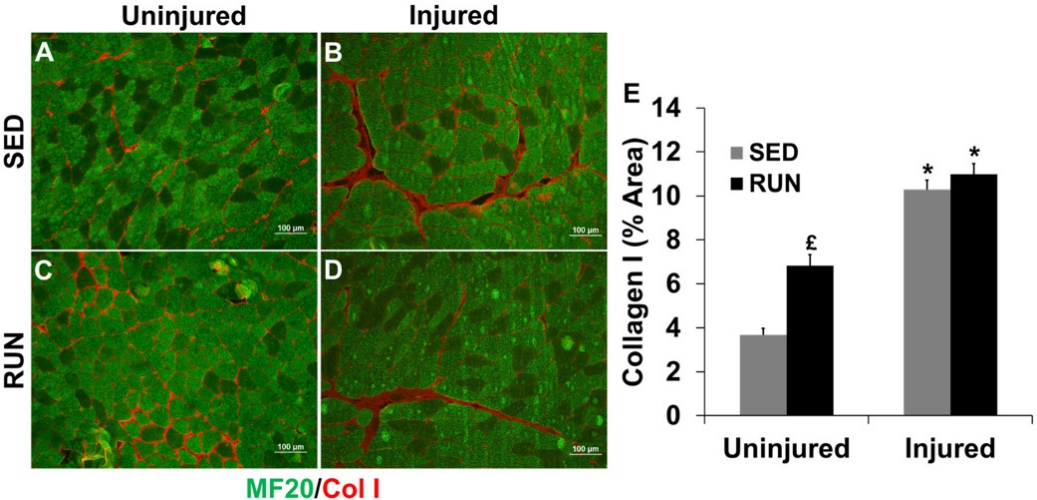
**Physical rehabilitation in the form of wheel running does not exacerbate injury related intramuscular collagen content.** Uninjured contralateral **(A,C)** and injured muscle **(B,D)** of SED and RUN groups, respectively were analyzed for intramuscular collagen content **(E)**. Scale bar = 100 μm. Only tissue within the injured muscle (not in the defect area) was included for analysis. Values are mean ± SEM. n = 3-6 muscles/group; * denotes ≠ uninjured (contralateral); £ denotes ≠ sedentary uninjured; p < 0.05.

**Figure 8:**
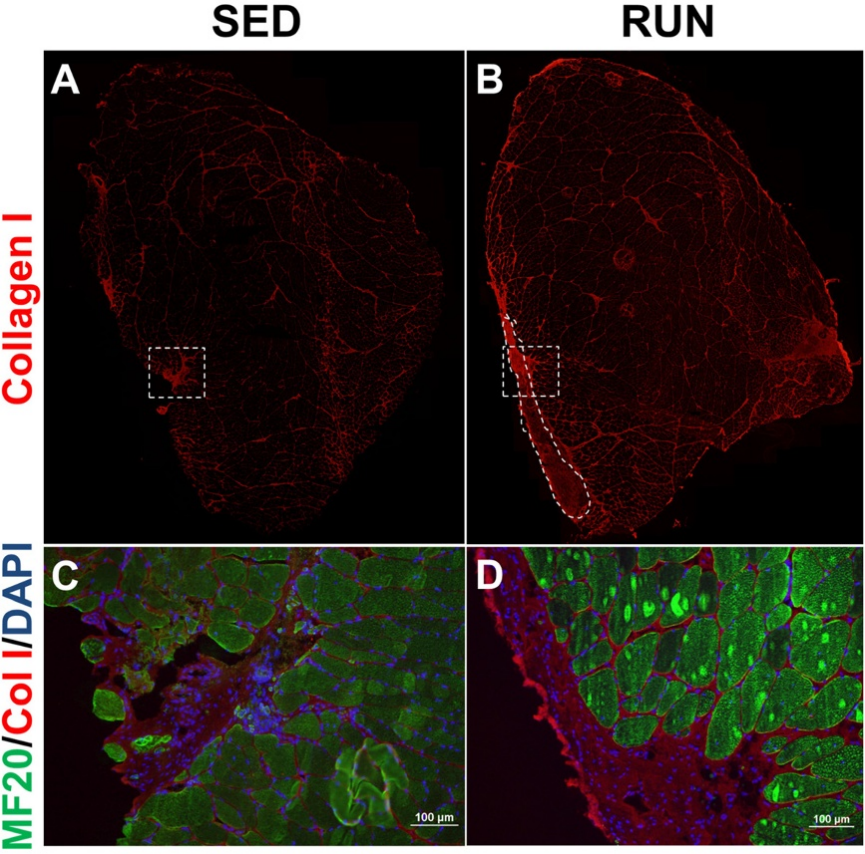
**Physical rehabilitation in the form of voluntary wheel running causes the development of a fibrotic scar in the defect area of the injured muscle.** Whole TA muscle cross-sections of the injured muscle of the SED **(A)** and RUN **(B)** groups are presented. White dashed line illustrates the formation fibrotic scar in the injured muscle of the RUN group **(B)**. White dashed boxes indicate the approximate region where images were taken in the defect area of the SED **(C)** and RUN **(D)** groups. No muscle regeneration was observed in either group.

The percent collagen I exclusively within the remaining muscle was calculated to examine the extent of collagen deposition due to injury and/or running. The uninjured muscle of the RUN group had ~40% higher collagen I than the SED group (p ≤ 0.05, Figure 7C). There were no differences in the intramuscular collagen content between the injured muscles (Figure 7A-B). However, the injured muscle of either group had ~ 50% more collagen deposition compared to the respective uninjured muscles (p ≤ 0.005). Qualitatively, there was increased collagen deposition (fibrotic scar) in the defect area of the RUN group than the SED group (Figure 8A-B) with no muscle fiber regeneration in either group (Figure 8C-D).

### Acute gene expression

**Figure 9:**
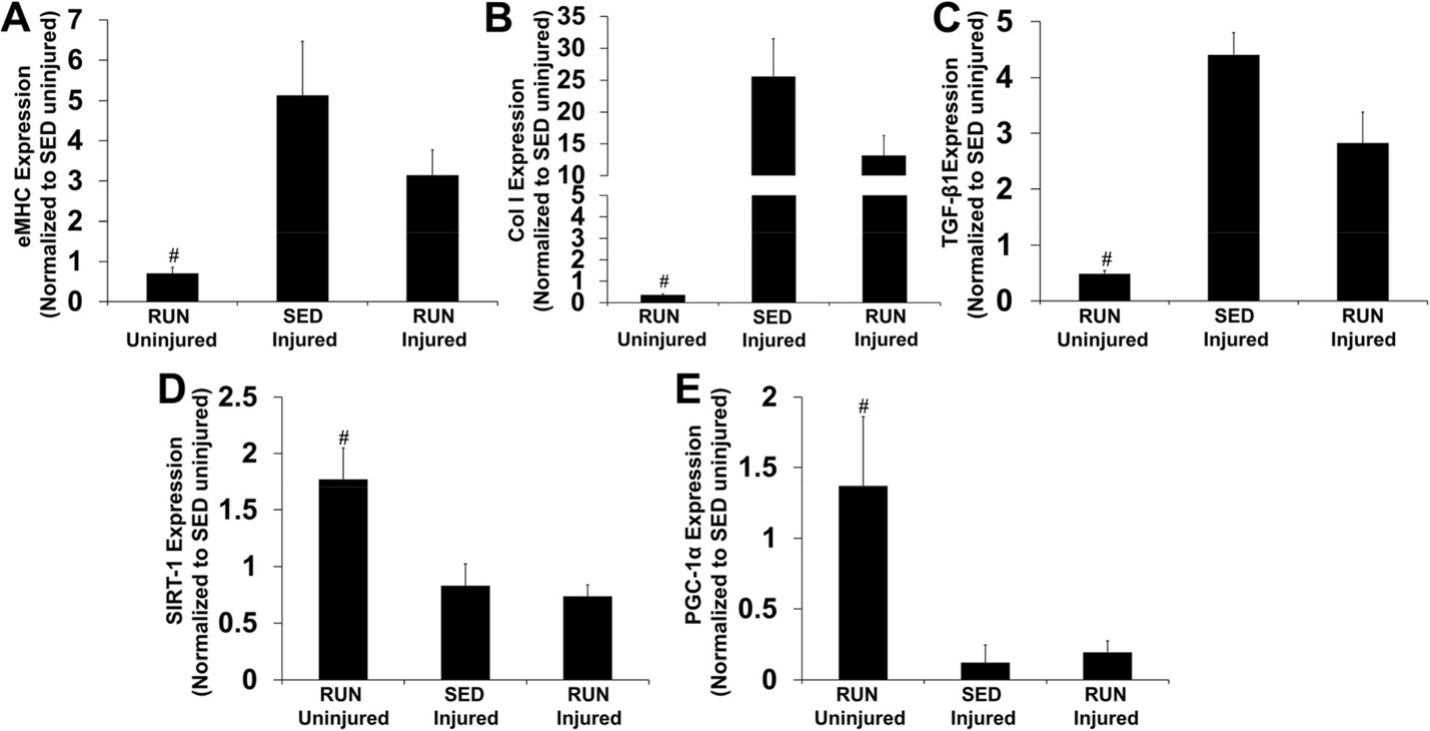
**Gene expression of myogenic and fibrotic markers is up regulated, while metabolic markers are down regulated in the injured muscle.** TA muscles from SED and RUN (one week of running) injured muscles were harvested two weeks post-injury. Tissue samples comprised of defect area and the remaining muscle were assayed for gene expression of **A)** Embryonic heavy chain myosin (eMHC), **B)** Collagen I (Col I), **C)** Transforming growth factor-β1 (TGF-β1), **D)** Silent mating type information regulation 2 homolog-1 (SIRT-1) and **E)** Peroxisome proliferator-activated receptor gamma co-activator 1 alpha (PGC-1α). Note: All gene expression data was normalized to SED uninjured. Values are mean ± SEM. n = 3-5 muscles/group; # denotes ≠ injured; p < 0.05.

To gain insight into the acute effects of wheeling running on the injured muscle, the gene expression of myogenic (eMHC), fibrotic (Collagen I, TGF-β1), and metabolic markers (SIRT-1, PGC-1α) was analyzed after one week of running (i.e., two weeks post-injury). The myogenic (Figure 9A) and fibrotic marker(s) (Figure 9B-C) were up-regulated in the injured muscle, while metabolic markers were down-regulated in the injured muscles when compared to uninjured muscle of the RUN group (Figure 9D-E).

## Discussion

In the absence of a definitive regenerative therapy, physical rehabilitation of the remaining muscle mass is often the standard of care for VML. The specific objectives of this study were to examine the functional and histomorphological adaptations in the injured muscle to physical rehabilitation. The primary findings of the study are that physical rehabilitation in the form of voluntary wheel running promotes ~ 17% improvement in maximal isometric torque, and a ~ 13% increase in weight of the injured muscle, but it did so without significant morphological adaptations (e.g., no hypertrophy and hyperplasia). These improvements reflect a ~31% recovery of the functional deficit in this VML model that is on par with functional benefits observed following the transplantation of decellularized ECM [6].

The general mechanism of functional recovery (T_max_) of VML injured muscle after physical rehabilitation (i.e., voluntary wheel running) was investigated. Running activity has been shown to foster regeneration of injured muscle [5, 32, 33] and promote hypertrophy (i.e., increased protein synthesis or muscle weight) in muscle grafts [34, 35]. However, in this study running did not result in an increase in muscle fiber number (hyperplasia) or cross-sectional area (hypertrophy) and did not increase embryonic myosin heavy chain expression acutely. Wheel running did up-regulate genes involved in mitochondrial biogenesis (SIRT-1, PGC-1α), but only in uninjured muscles. Instead of muscle regeneration, a greater deposition of fibrous tissue preceded by an up-regulation of pro-fibrotic genes (Collagen I, TGF-β1) was observed in the defect area and therefore, it is plausible that wheel running related functional improvements were due to improved force transmission but not generation. Previously, using the same VML model, we have shown a fibrotic scar formed due to remodeling of an extracellular matrix derived scaffold promoted functional recovery 16 weeks post-injury [6]. Thus, it would appear that extracellular matrix deposition in the defect area of VML injured muscle may be a positive adaptation for optimal transmission of force generated by the remaining muscle tissue.

Strengthening of synergist muscles can partially compensate for the loss of function due to VML injury. Compensatory hypertrophy after synergist muscle ablation is a well-described adaptation [36–39]. In the anterior compartment, whole tibialis anterior muscle ablation has been shown previously to promote a 20 - 25% increase in maximal force of the EDL muscle over a one-month period [40–42]. Similarly, herein a partial VML in the TA muscle resulted in a ~20–22% increase in EDL muscle weight and strength by eight weeks post-injury in sedentary rats. However, wheel running attenuated the compensatory response of the EDL as the TA muscle gained strength. Two clinical ramifications of these findings are 1) the net gain in function of the injured muscle unit may reflect the strengthening of the injured musculature, but the progressive weakening of the synergists and 2) physical rehabilitation may mitigate secondary joint complications that arise from chronic synergist muscle functional imbalances [43, 44].

The prolonged pathophysiology in the remaining musculature following VML is not well understood, raising questions regarding appropriate physical rehabilitation regimen. A consistent observation made among VML studies in our lab group is the continued presence of centrally located nuclei in the injured muscle fibers, indicating chronic injury and remodeling [6, 7]. Wheel running resulted in a two-fold increase in the number of centrally located nuclei in the remaining (injured) muscle. It is plausible that the already overloaded injured TA muscle is further damaged due to repetitive loading during wheel running, and that a physical rehabilitation regimen imposing greater mechanical loads may be deleterious to long-term functional outcomes. However, though limited to this rat model and these experimental conditions, these findings highlight that an improved understanding of the pathophysiology of VML will be important in prescribing an appropriate regimen of physical rehabilitation for this indication.

Voluntary wheel running allows the animal to determine the frequency, intensity, and volume of activity and is a convenient and clinically relevant form of physical rehabilitation. Since, voluntary wheel running stimulates low resistance aerobic exercise it does not impose sufficient load on the TA muscle to cause morphological adaptations as seen in this study. Hence, future work will examine resistance (e.g., ladder climbing) and/or higher intensity training (e.g., treadmill running) regimens, amongst others. Physical rehabilitation can start within days or weeks following surgery. Initiation of wheel running one week post-injury during the early phase of healing may not reflect all clinical scenarios. Therefore, optimal timing of initiating rehabilitation needs to be investigated. Lastly, TA muscle is a non-load bearing muscle, therefore future work is needed to examine similar changes in load bearing muscles.

## Conclusions

This is the first pre-clinical study to demonstrate improvement in functional performance of non-repaired VML injured muscle with physical rehabilitation in the form of voluntary wheel running. This study provides information for the first time on the basic changes in the VML injured muscle with physical rehabilitation, which may aid in the development of appropriate physical rehabilitation regimen(s).

## Authors’ information

Department of the Army, Extremity Trauma and Regenerative Medicine, Institute of Surgical Research, 3650 Chambers Pass, JBSA Ft Sam, Houston, TX 78234-7767, USA.
